# Recent Developments in Bio-Ink Formulations Using Marine-Derived Biomaterials for Three-Dimensional (3D) Bioprinting

**DOI:** 10.3390/md22030134

**Published:** 2024-03-16

**Authors:** Zied Khiari

**Affiliations:** National Research Council of Canada, Aquatic and Crop Resource Development Research Centre, 1411 Oxford Street, Halifax, NS B3H 3Z1, Canada; zied.khiari@nrc-cnrc.gc.ca

**Keywords:** 3D bioprinting, marine biomaterials, bio-ink, polysaccharides, proteins

## Abstract

3D bioprinting is a disruptive, computer-aided, and additive manufacturing technology that allows the obtention, layer-by-layer, of 3D complex structures. This technology is believed to offer tremendous opportunities in several fields including biomedical, pharmaceutical, and food industries. Several bioprinting processes and bio-ink materials have emerged recently. However, there is still a pressing need to develop low-cost sustainable bio-ink materials with superior qualities (excellent mechanical, viscoelastic and thermal properties, biocompatibility, and biodegradability). Marine-derived biomaterials, including polysaccharides and proteins, represent a viable and renewable source for bio-ink formulations. Therefore, the focus of this review centers around the use of marine-derived biomaterials in the formulations of bio-ink. It starts with a general overview of 3D bioprinting processes followed by a description of the most commonly used marine-derived biomaterials for 3D bioprinting, with a special attention paid to chitosan, glycosaminoglycans, alginate, carrageenan, collagen, and gelatin. The challenges facing the application of marine-derived biomaterials in 3D bioprinting within the biomedical and pharmaceutical fields along with future directions are also discussed.

## 1. Introduction

3D bioprinting is an emerging and advanced computer-aided additive manufacturing technology that precisely dispenses biomaterials, layer-by-layer, to build complex 3D structures [1,2]. 3D bioprinting enables the fabrication of a number of constructs, such as artificial tissues, artificial organs, cells, and blood vessels [3], which could represent a possible solution to the organ shortage crisis facing transplant medicine [4]. In addition, 3D bioprinting offers tremendous opportunities in the pharmaceutical field, including in vitro/in vivo modeling, the development of drug delivery systems along with drug screening, and the development of novel drug discovery systems [4,5].

Current 3D bioprinting technologies comprise three major categories: jet-based, extrusion-based, and vat polymerization-based bioprinting [6]. Jet-based bioprinting is one of the pioneering 3D printing technologies [7] which shares significant similarities to conventional 2D jet printing [8]. Jet-based bioprinting comprises inkjet, micro-valve, laser-assisted, and acoustic bioprinting techniques [9]. Extrusion-based bioprinting originated from fused deposition modeling technology, which relies on material fusion followed by extrusion and deposition [6]. Extrusion-based bioprinting could be categorized into three groups, pneumatic-, screw- or piston-driven, depending on the bio-ink-dispensing system [10]. Vat polymerization-based bioprinting, which includes stereolithography, digital light processing, and two-photon polymerization, relies on the polymerization of a liquid resin after exposure to a light source of specific wavelength [11].

Bio-inks, the soft biomaterials comprising living cells and/or biologically active molecules, that are used in 3D bioprinting processes [12,13], play a major role in the successful production of 3D constructs [14]. In this regard, the process of bioprinting is heavily relying on the deposition of bio-inks into designed shapes generated through computer-aided design (CAD) models and, therefore, the bio-inks need to possess a number of key properties, including printability (i.e., viscoelastic properties), printing fidelity, mechanical integrity, and biocompatibility [13,14]. It is worth noting that in the biomedical field, 3D bioprinting differs in practice from 3D printing [15]. 3D bioprinting refers to printing using living cells and/or biologically active molecules which are incorporated within the bio-ink. 3D printing, on the other hand, refers to the fabrication of complex, non-cellular networks (i.e., scaffolds) that are seeded with living cells and/or biologically active molecules post-printing [16,17].

Owing to their high mechanical strength, polycaprolactone (PCL), poly-L-lactic acid (PLA), and poly-lactic-co-glycolic acid (PLGA), are currently the most commonly used synthetic materials for bioprinting [14,18]. However, since PCL, PLA, and PLGA can only be printed when melted at higher temperatures or dissolved in organic solvents, these materials are unsuitable for printing living cells [12]. Due to their natural origin, marine-derived biomaterials (such as chitosan, hyaluronic acid, chondroitin sulfate, dermatan sulfate, alginate, carrageenan, collagen, and gelatin) have recently received a great deal of attention, since they possess enormous potential and hold promise to replace synthetic materials in bioprinting [14].

The aim of the present review is to provide a general overview of 3D bioprinting. Marine-derived biomaterials, that could be used as bio-inks, including polysaccharides (chitosan, glycosaminoglycans [hyaluronic acid, chondroitin sulfate and dermatan sulfate], alginate and carrageenan) as well as proteins (collagen and gelatin) are the central focus of this review. The prospects and challenges of the application of marine-derived biomaterials in 3D bioprinting are also discussed.

## 2. Overview of 3D Bioprinting Technologies

3D bioprinting is an additive manufacturing process (in contrast to the conventional subtractive manufacturing that is based on the removal of materials), that relies on the precise deposition of biomaterials, in micrometer scale, in order to build complex 3D structures [19]. To date, there are three major bioprinting techniques: jet-based, extrusion-based, and vat polymerization-based bioprinting. However, no single bioprinting technique is able to produce all scales and complexities of target structures [20], and each of these bioprinting techniques have specific advantages, drawbacks and limitations (Table 1). It is, however, worth mentioning that all these bioprinting technologies still possess great potential for further developments.

### 2.1. Jetting Bioprinting

Jet-based bioprinting is a non-contact technique that enables the production of 3D structures using picolitre bio-ink droplets layered onto a substrate [58]. Several factors affect both the printability and cell viability in jet-based bioprinting. For instance, Ng et al. [59] investigated the influence of bio-ink properties on printing performance and cell health. Their findings indicated that a higher bio-ink viscoelasticity leads to a better stabilization of droplet filaments before rupturing from the nozzle orifice. In addition, increasing the bio-ink’s viscosity promoted droplet deposition on the substrate surface and improved the accuracy of the droplet deposition. When it comes to cell viability, results from the same study suggested that printing with viscous bio-inks helps in maintaining higher cell viability [59].

Xu et al. [60] investigated the effect of cell concentration in the bio-ink on the droplet formation in relation to jet-based bioprinting process. Their results showed that increasing the cell concentration in the bio-ink is inversely correlated with the droplet size and velocity (i.e., increasing the cell concentration decreases the droplet size and velocity) and is positively correlated with the breakup time (i.e., increasing the cell concentration increases the breakup time).

Other physical factors can also affect the cell viability in jet-based bioprinting, including shear stress [7,61] as well as droplet impact velocity and volume [62]. For instance, Blaeser et al. [61] carried out a study to investigate the effect of shear stress levels on cell viability and proliferation potential. Their outcomes demonstrated that short-time exposure to high levels of shear stress has an immediate negative effect on cell viability and can further lead to long-term changes in the proliferation potential of the unaffected cells. The findings of the same study suggested that cells can be printed without side effects below a specific shear stress threshold [61]. Regarding the effect of droplet impact velocity and droplet volume on cell viability, Ng et al. [62] reported that increasing cell concentration in the bio-ink leads to a slower impact velocity which, in turn, results in a higher viability of the printed cells. The outcomes for the same study also indicated that a minimum droplet volume of 20 nL per spot reduces the cell damage that is typically induced by evaporation and preserves the viability of the printed cells within a printing duration of 2 min [62]. These findings suggest that in order to maintain viability and proliferation, the droplet impact velocity and droplet volume must be carefully controlled in sub-nanoliter bioprinting [62].

**Figure 1:**
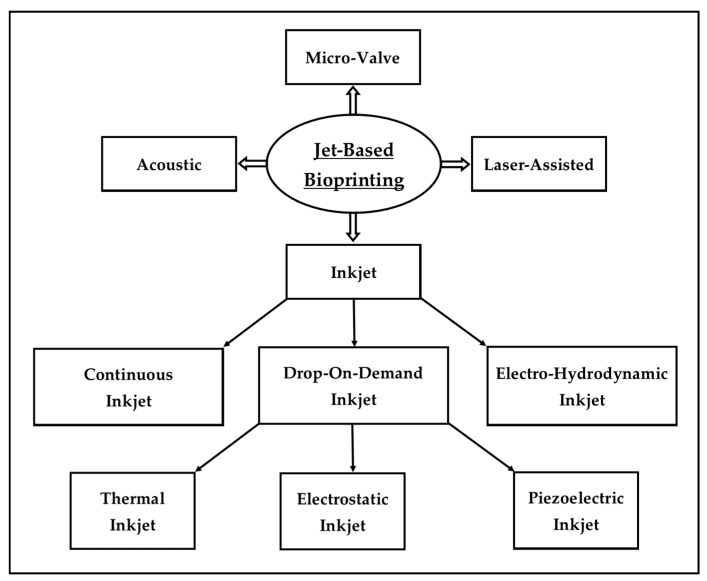
Illustration of the different classes of jet-based bioprinting.

In general, there are four major classes of jet-based bioprinting (Figure 1): inkjet, micro-valve, acoustic, and laser-assisted [9,23].

#### 2.1.1. Inkjet Bioprinting

Inkjet bioprinting is a nozzle-based printing process, in which droplets are formed then selectively and precisely deposited onto a substrate to build a 3D structure [63]. There are three classes of jet-based bioprinting: continuous inkjet, drop-on-demand inkjet, and electro-hydrodynamic jet bioprinting (Figure 1). Among the three groups, the drop-on-demand inkjet is the most commonly used technique and consists of either thermal, piezoelectric, or electrostatic inkjet bioprinting [23].

The low-cost, high printing speed as well as the greater cell viability are the main advantages of inkjet bioprinting; however, this bioprinting technique is not suitable for high viscosity bio-inks containing high cell density [20]. Another disadvantage associated with this technique is related to the “settling effect”, which is observed when the cells settle in the cartridge over time which, in turn, leads to increasing the bio-ink’s viscosity and the clogging of the printer head [64].

#### 2.1.2. Micro-Valve Bioprinting

Micro-valve bioprinting is a printing process in which droplets are deposited in a controlled and layer-by-layer manner [65]. It utilizes an electromechanical valve (i.e., a solenoid) to control the flow rate of the bio-ink [66]. This bioprinting method is believed to print at high resolutions as well as high cell viability and printing speed [28]. However, the major challenge with micro-valve bioprinting is associated with the maximum bio-ink viscosity that allows the reliable printing of high cell concentration mixtures without negatively affecting the cell viability [65].

#### 2.1.3. Laser-Assisted Bioprinting

Laser-assisted bioprinting relies on the laser-induced forward transfer (LIFT) effect, which enables the deposition of materials in solid or liquid phases [19]. Studies indicated that live cells can successfully be deposited using laser-induced forward transfer-based techniques [67,68]. However, it was recognized that laser-assisted bioprinting reduces the cell survival rate, which is attributed to thermal damage induced by nanosecond-scale laser exposure [19].

A number of critical parameters significantly affect the LIFT process [69]. These parameters include the fluence (i.e., the energy of the laser pulse per unit area), the donor-receiver substrate distance, the characteristics of the pulse, the laser spot size, as well as the materials used in the LIFT process [69]. Despite having several advantages, the LIFT process still faces important challenges. For instance, shockwave formation is among the major drawbacks of the LIFT process [69]. Shockwaves are the result of a spike in temperature and pressure within the small volume and are responsible for stopping and disintegrating the pixel when they reach the receiver [69,70]. Proposed strategies to reduce or eliminate shockwaves in LIFT bioprinting include operating in vacuum or low-pressure environments, the application of laser beam shaping techniques, as well as the use of femtosecond laser pulses [69]. Other factors that affect the LIFT bioprinting comprise the strength of bond between the deposits and the poor adhesion between the deposit material and the receiver substrate as recently reviewed by Das et al. [69].

#### 2.1.4. Acoustic Bioprinting

The acoustic droplet ejection technology is an ultrasound-assisted bioprinting method. As described by Jentsch et al. [71], the acoustic droplet ejection bioprinting process generates a droplet, using ultrasonic frequencies, at an air–liquid interface from an open reservoir containing the bio-ink. In the case of acoustic bioprinting, the droplets are ejected from an open pool rather than a nozzle as with inkjet bioprinting [23]. The size of the droplet can be controlled by manipulating the frequency of the ultrasound signal since the droplet size is inversely proportional to the frequency of the ultrasound signal [71].

### 2.2. Extrusion Bioprinting

In extrusion-based bioprinting, the systems dispense continuous filaments of a material through a micro-nozzle to build the 3D structure [58]. Extrusion bioprinting is by far the most widely used bioprinting method, owing to its simplicity, diversity, and predictability [19]. Unlike jet-based bioprinting, extrusion bioprinting is capable of handling higher cell densities and allows the use of highly viscous biomaterials; however, this advantage comes with the tradeoff of a lower speed [19,58].

**Figure 2:**
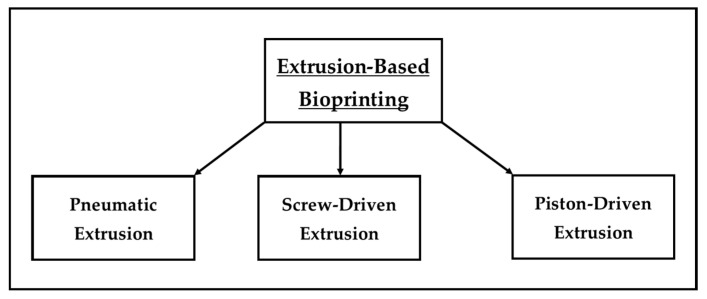
Illustration of the three classes of extrusion-based bioprinting.

There are three common classes of extrusion-based bioprinting (Figure 2): pneumatic extrusion (driving force: pressure), piston-driven extrusion (driving force: vertical mechanical force), and screw-driven extrusion (driving force: rotational mechanical force) [19]. Among the three classes of extrusion-based bioprinting, pneumatic extrusion is the most commonly used method [72].

Several factors affect the printability in extrusion-based bioprinting. It has been reported that the bio-ink’s viscosity can have both positive and negative impacts on printability in extrusion-based bioprinting [73]. For instance, higher bio-ink’s viscosity usually results in higher filament fidelity and structural integrity, which is attributed to the fact that it is harder for viscous bio-inks to flow and spread [73]. Also, cells within viscous bio-inks tend to be homogeneously distributed, and the higher viscosity prevents the sedimentation of the cells [73]. Regarding the negative impacts of bio-ink’s viscosity on printability, it has been observed that higher viscosity adversely affects extrudability, which is associated with the higher-pressure requirements during the extrusion process [73].

Bio-ink’s viscoelasticity also affects the printability in extrusion-based bioprinting. In this regard, elastic bio-inks have been reported to possess inferior printability, which is due to the swelling of the extruded filament as a result of the deformation, also known as the Barus effect [74]. On the other hand, the bio-ink needs to possess superior gelation characteristics to retain its shape without spreading [13].

Another critical physical factor that affects the printability in extrusion-based bioprinting is the surface tension of the bio-ink [13,73]. In this respect, the bio-ink needs to possess low surface tension properties in order to avoid attachment on the surface of the nozzle tip, which, in turn, facilitates the droplet formation and subsequent filament extrusion [13].

When it comes to the influence of extrusion-based bioprinting on cell viability, it is well-known that the cells are subjected to several mechanical forces. Among these forces, shear stress is the most critical one as it is the leading cause of cell damage and/or death [75]. Shear stress is also strongly related to the bio-ink viscosity. Several studies demonstrated that highly viscous bio-inks require high pressure during extrusion, which, in turn, increases the shear stress and can lead to the rupture of the cell membrane [76,77].

### 2.3. Vat Polymerization Bioprinting

Vat polymerization-based bioprinting uses light to cure and harden liquid photosensitive resins [78,79]. Ultraviolet (UV) radiation is often used for the photopolymerization process; however, longer wavelengths of light, from visible to near infrared, have been recently used in order to address a number of issues associated with UV radiation [11,80].

In vat polymerization-based bioprinting, the light source is the key factor affecting both the cell viability and the printing speed [81]. In the photopolymerization process, unsaturated molecules in the bio-ink undergo a conversion into solid macromolecules through photonic energy [82]. UV light, and to a lesser extent, visible light, as well as near infra-red (NIR) femtosecond laser are often used to induce polymerization [81,83]. Since UV light can lead to genetic modification and cell death, the recent advances and tendencies in vat polymerization-based bioprinting is to use visible light in order to overcome the challenges facing cell viability that are observed with UV light-based photopolymerization [81]. Regarding the printing speed, it has been reported that decreasing the exposure period reduces the overall printing time and, thus, increases the printing speed [81]. However, the major drawback with this procedure is a lower printing resolution due to incomplete photopolymerization [81]. Increasing the light intensity, on the other hand, can lead to a higher crosslinking, but can also affect the overall photopolymerization reaction and potentially lead to cell damage and/or death [81,84].

A critical parameter that plays a major role in the light-based polymerization bioprinting is the photopolymer that is used in the bio-ink. Photopolymers are materials that, when exposed to light, undergo photochemical reactions [85]. Photopolymers consist of monomers, oligomers, polymers, or mixtures of all these forms [85]. In 3D vat polymerization-based bioprinting, the most widely used photopolymers are reactive multifunctional monomers and/or oligomers together with a photo-initiator [85]. The main issue with current photopolymers in 3D bioprinting is the internal shrinkage of the cured bio-ink, which, in turn, leads to the distortion and loss of shape of the produced 3D structures [86]. In addition, bioprinting of soft tissues by photopolymerization-based approaches is challenging [87]. Marine-derived biomaterials, such as hyaluronic acid and gelatin, could offer the possibility to produce 3D bioprinted structures without compromising their mechanical properties and fidelity. For example, Zhang et al. [88] generated a photopolymerized 3D printed scaffold for postsurgical tumor treatment using gelatin-based bio-ink combined with Pt(IV) prodrug, as the photo-initiator. Wang et al. [87] proposed a method for high-performance bioprinting using hyaluronic acid methacrylate mixed with photo-activated groups.

**Figure 3:**
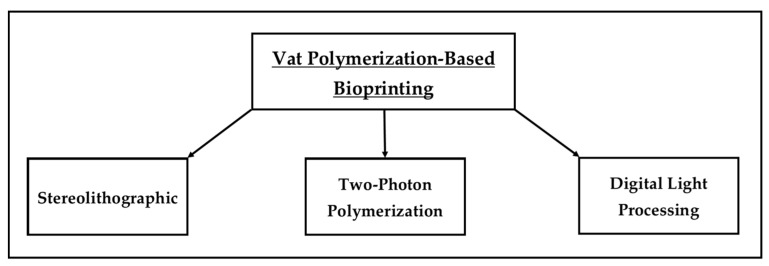
Illustration of the three classes of vat polymerization-based bioprinting.

There are three major classes of vat polymerization-based bioprinting (Figure 3): stereolithographic bioprinting, two-photon polymerization, and digital light processing.

#### 2.3.1. Stereolithographic Bioprinting

Stereolithography bioprinting is a versatile technology that offers the possibility of designing a large number of structures in a wide range of scales, spanning sub-microns to decimeters [14]. The stereolithography bioprinting technique relies on sending a laser beam to a polymer that interacts with light [3,89]. In this regard, a structure is formed through the deposition of photosensitive polymer, layer-by-layer, until obtaining the desired structure [3,90]. A photosensitive resin in a vat is needed in stereolithography bioprinting, and the crosslinking of material is achieved through point-by-point scanning [91]. The light intensity in stereolithography bioprinting is controlled through digital micromirror arrays [92].

Stereolithography offers higher printing quality, speed, and cell viability compared to both jet- and extrusion-based bioprinting. However, along with the scarcity of biocompatible materials with suitable properties for stereolithography bioprinting [14], the use of UV light in the polymerization stage could cause serious health issues, including DNA damage which could, subsequently, lead to cancer [19].

#### 2.3.2. Two-Photon Polymerization

Two-photon polymerization is a photochemical 3D microfabrication process that uses light, which initiates two-photon polymerization through two-photon absorption and subsequent polymerization [93,94]. Two-photon polymerization-based bioprinting is a nozzle-free technology that is effective in building 3D structures having a resolution of 100 nm or higher [93]. However, bioprinting at the nano-scale resolution is not practical due to the extremely low productivity. Bioprinting using the two-photon polymerization process in the micron resolution range (tens of micrometers) is more useful for manufacturing 3D microstructures. Two-photon polymerization has been found to be useful for the fabrication of scaffolds, nano- and micro-robots, as well as microfluidics [83]. Unlike stereolithography-based bioprinting in which the formation of objects is based on a layer-by-layer manner [95], two-photon polymerization technology is able to print complex 3D constructs without any geometrical limitations [49,50].

#### 2.3.3. Digital Light Processing

Digital light processing-based bioprinting is a fast and robust bio-fabrication technique [96] that is capable of producing 3D structures with complex geometries and high resolution [97]. The process, which uses a digital micromirror device (DMD) or a liquid crystal display (LCD), relies on projecting the desired image or pattern on the surface of the bio-ink, which in turn results in localized photopolymerization and the formation of sophisticated objects [96,97,98]. The LCD is based on transmissive planar light patterns, while the DMD relies on the reflective processing of digital patterns [99]. LCD is made of layers of liquid crystals and electrodes inserted between polarizing filters [100]. By applying electrical current to the LCD, molecular rearrangements block the light from crossing the display [101], which shines directly onto the uncured resin [102]. In the DMD, there are two modes: ON and OFF. In the ON state, the light is reflected to the projection lens, and on the OFF state, light is reflected to the absorbing element [103]. By controlling both states (i.e., ON and OFF), the selective reflection of incoming light can be obtained [100,103]. It has been reported that images produced by the LCD-based mode are sharper and possess superior quality at lower intensities, while those obtained through a DMD-based mode are lighter, portable, and efficient in delivering high intensity light patterns [96].

Compared to extrusion-based bioprinting, digital light processing offers faster printing speeds since the 3D structure formation is based on the layer-by-layer approach rather than the deposition of a linear filament [104]. When compared to laser-based stereolithography bioprinting, digital light processing seems to be faster and more efficient [98].

## 3. Marine-Derived Biomaterials for Bio-Ink Formulations

Biomaterials play a critical role in 3D bioprinting. Recent advances in marine research indicated that marine organisms along with fisheries and aquaculture processing waste represent an abundant source of biopolymers [105] that could be suitable for 3D bioprinting. Marine biomaterials have received a great deal of attention in recent years as bio-ink components for 3D bioprinting applications [14]. Chitosan, glycosaminoglycans [hyaluronic acid, chondroitin sulfate, and dermatan sulfate], alginate, carrageenan, as well collagen and gelatin are among the most commonly used marine-derived biomaterials in 3D bioprinting.

### 3.1. Polysaccharides-Based Marine-Derived Biomaterials

#### 3.1.1. Chitosan

**Figure 4:**
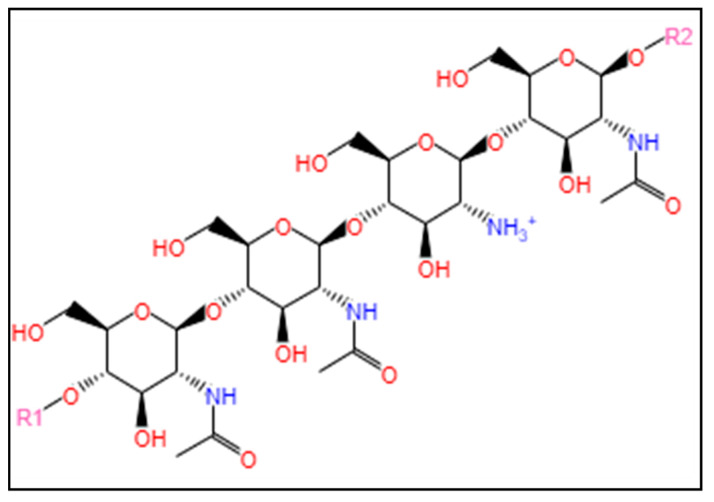
Chemical structure of chitosan. The structure was obtained from the MetaCyc database using the keyword “chitosan” for the compound search [110].

Chitosan is the deacetylated derivative of chitin—the second most abundant natural polysaccharide after cellulose [106]. Both chitin and chitosan belong to a family of linear polysaccharides comprising varying amounts of β-(1-4) linked residues of N-acetyl-glucosamine (N-acetyl-2 amino-2-deoxy-D-glucose) and glucosamine (2-amino-2-deoxy-D-glucose) residues [107]. The chemical structure of chitosan is shown in Figure 4. In the marine world, chitin is largely found in the exoskeleton of crustacea (e.g., shrimp, lobster, crab, etc.). The high levels of acetylated groups, rigid structures, and poor solubility of chitin limit its industrial application [108]. Chitosan, on the other hand, possesses greater solubility than chitin and is biocompatible and biodegradable [109].

There are two main preparation methods for chitosan; chemical and enzymatic processes. In the chemical process of chitosan extraction from marine sources, the exoskeleton of crustacea undergoes decalcification, deproteinization, and decolorization steps in order to produce chitin, which is subsequently treated with a concentrated alkaline solution (typically 40–50% NaOH) to remove the acetyl groups and obtain chitosan [108]. Several factors affect the chemical process, including the concentration of the alkaline solution, the reaction time, the process temperature, and the ratio of chitin/alkaline solution [109]. Despite the simplicity of the chemical chitosan preparation method, it still requires high energy input and generates harmful chemical effluents that need to be treated before discharge.

In the enzyme-assisted process, the deacetylation of chitin to chitosan is achieved enzymically using chitin deacetylase. Despite the potential of the enzyme-assisted process as an alternate greener method that could replace the chemical methods, it still faces issues, especially in selecting, growing, and isolating suitable enzyme (chitin deacetylase)-producing microorganisms [108].

#### 3.1.2. Glycosaminoglycans

Glycosaminoglycans are linear polysaccharides abundantly present in the extracellular matrices of animals. Glycosaminoglycans are polymers consisting of repeating *O*-linked disaccharide units which are generally sulfated, except for hyaluronic acid [111]. Glycosaminoglycans are usually found as part of proteoglycans, where the polysaccharide side chains are covalently bound to protein cores [112]. Glycosaminoglycans from marine environments have been found to be different from those of terrestrial origins, and the differences are related to the molecular weights and the sulfation levels [113].

Hyaluronic acid, chondroitin sulfate, and dermatan sulfate have been reported to be the most suitable glycosaminoglycans in bio-ink formulation for 3D bioprinting [14].

**Figure 5:**
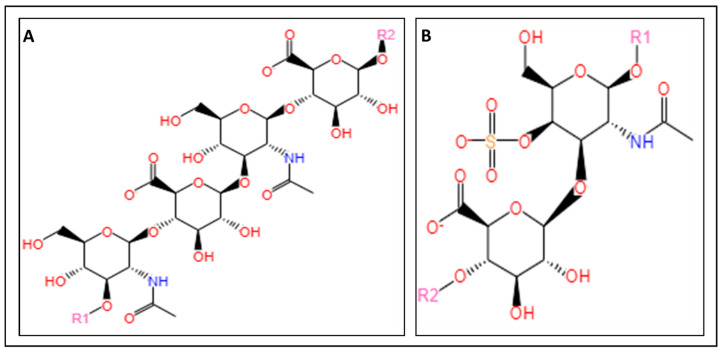
Chemical structures of hyaluronic acid (**A**) and chondroitin sulfate (**B**). The structures were obtained from the MetaCyc database using the keywords “hyaluronan” [119] and “chondroitin sulfate” [120] for the compound search.

Hyaluronic acid is a long, unbranched, non-sulfated polysaccharide, composed of repeating disaccharides of d-glucuronic and *N*-acetyl-d-glucosamine [114]. Hyaluronic acid is negatively charged, highly hydrophilic, and forms a viscous network at high molecular weights [115]. The chemical structure of hyaluronic acid is shown in Figure 5A.

Chondroitin sulfate, on the other hand, is a homo-polymeric glycosaminoglycan [i.e., a polymer made from many copies of a single repeating unit [116]] comprising a single repeating disaccharide unit of -4GlcAβ1-3GalNAcβ1-, where GlcA and GalNAc represent d-glucuronic acid and *N*-acetyl-d-galactosamine, respectively [117]. The disaccharide units of d-glucuronic acid and *N*-acetyl-d-galactosamine within chondroitin sulfate are sulfated at different positions with various combinations [118]. The chemical structure of chondroitin sulfate is shown in Figure 5B.

The chemical composition of dermatan sulfate is the same as that of chondroitin sulfate; however, the only difference is that some of the glucuronic acid residues undergo epimerization to form iduronic acid [111].

Since glycosaminoglycans are usually found as part of proteoglycans, the extraction method relies on a hydrolysis step (either chemically or enzymatically) to liberate glycosaminoglycans from the protein cores. In the chemical process, the animal or fish tissue materials are chemically digested through an alkaline hydrolysis (using high concentrations of either NaOH, urea, or guanidine HCl), followed by the elimination of proteins and the recovery and purification of the target glycosaminoglycan [121]. In the enzyme-assisted process, the animal or fish tissue material is first hydrolyzed with a suitable protease to liberate glycosaminoglycans from the protein cores. Glycosaminoglycans are then separated from the protein hydrolysates (or peptide mixture) through ultrafiltration (typically using a 10 kDa cutoff membrane). The recovered glycosaminoglycan fraction is subsequently isolated and further purified [14,121].

#### 3.1.3. Alginate

**Figure 6:**
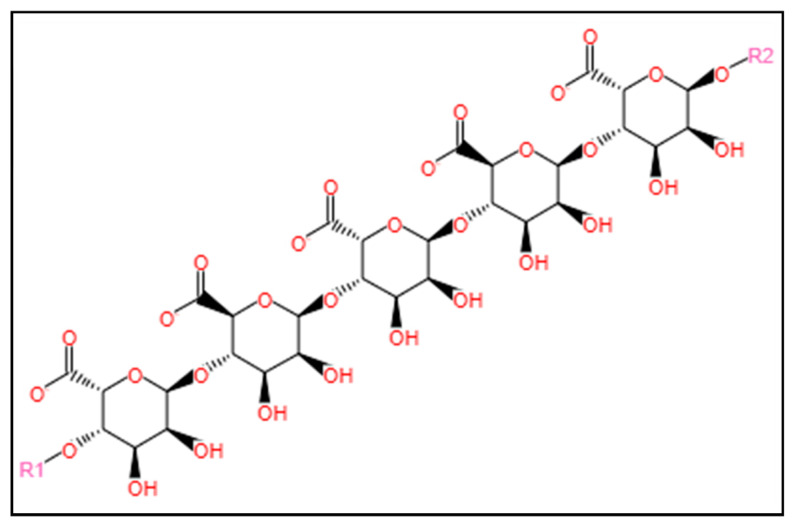
Chemical structure of alginate. The structure was obtained from the MetaCyc database using the keyword “alginate” for the compound search [123].

Alginate is an edible linear anionic heteropolysaccharide, composed of 1,4-linked β-D-mannuronic acid and 1,4 α-l-guluronic acid residues [122]. The chemical structure of alginate is shown in Figure 6. In marine environments, alginate is widely present in brown seaweed [14]. Due to its high gel-forming property, alginate has found applications in several industries including food, textile, and biomedical fields [122].

The extraction of alginate from brown seaweed requires an initial treatment with a mineral acid to convert the salt of alginic acid into free alginic acid, followed by a neutralization step to form soluble sodium alginate [124]. The soluble alginate is then precipitated either into calcium alginate fiber or alginic acid gel using calcium chloride or mineral acid, respectively. The latter products are commonly converted into sodium alginate through a treatment with sodium carbonate [124].

#### 3.1.4. Carrageenan

Carrageenan is a water-soluble anionic sulfated polysaccharide present in marine red algae [11]. Carrageenan is made of alternating 3-O-substituted β-d-galactopyranosyl units and 4-O-substituted α-d-galactopyranosyl units [125].

**Figure 7:**
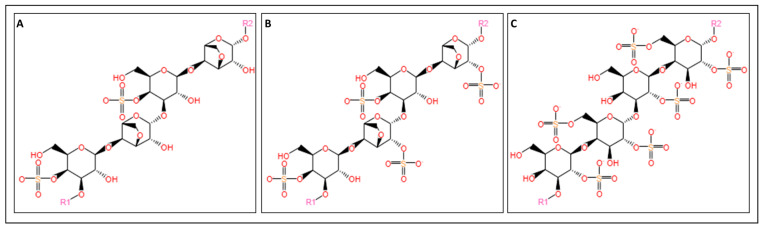
Chemical structures of carrageenans: (**A**) the structure of κ-carrageenan, (**B**) the structure of ι-carrageenan, and (**C**) the structure of λ-carrageenan. The structures were obtained from the MetaCyc database using the keywords “Kappa (κ)-carrageenan” [128], “Iota (ι)-carrageenan” [129], and “Lambda (λ)-carrageenan” [130] for the compound search.

The most industrially utilized carrageenans are Kappa (κ)-, Iota (ι)-, and Lambda (λ)-carrageenans [126], which have excellent stability and gel-forming properties. Kappa, Iota, and Lambda carrageenans (Figure 7) are characterized by the presence of either one, two, or three ester-sulfate groups per repeating disaccharide unit, respectively [127]. In both κ- and ι-carrageenans (Figure 7A and Figure 7B, respectively), the 4-O-substituted α-d-galactopyranosyl units contain an internal 3,6 ether bond—also called 3,6-anhydro-α-d-galactopyranosyl units [125].

*Chondrus*, *Eucheuma*, *Gigartina*, and *Hypnea* are the four major seaweed species from which carrageenan is industrially extracted [131]. The extraction of carrageenan starts with the drying of the seaweed to avoid degradation, followed by a thorough water-wash to remove impurities. The clarified seaweed is subsequently hydrolyzed using an alkaline treatment at high temperature, which liberates carrageenan from the seaweed cell wall. After the chemical hydrolysis, the carrageenan solution is further clarified and dried [132]. Several factors affect the chemical carrageenan extraction process, including temperature, pH, and process duration [131].

### 3.2. Protein-Based Marine-Derived Biomaterials

#### 3.2.1. Collagen

Collagen is the most abundant animal protein and is dominant in the extracellular matrix and connective tissues [133,134]. This superfamily of fibrous proteins is characterized by having each collagen chain containing repeating amino acid motifs (Gly–X–Y), in which X represents either proline or hydroxyproline and Y refers to any amino acids [135]. Collagen comprises three polypeptide chains (referred to as α-chains), which could be identical or different [136]. Fisheries processing by-products and wastes, which consist of heads, skins, trimmings, scales, and bones, contain large amounts of Type I collagen [105,137]. Type I collagen contains two identical α_1_-chains and one α_2_-chain, forming a right-handed triple helix structure [138]. The collagen triple helix structure is stabilized by proline and hydroxyproline though hydrogen bonding which, in turn, restricts rotation [139,140]. Collagen has found many applications in a variety of industries such cosmetic, biomedical, pharmaceutical, and nutraceutical fields.

The extraction of collagen is based on several steps. It starts with a pretreatment for the removal of fat, non-collagenous proteins, and partial hydrolysis of collagen crosslinks, followed by collagen extraction, and further purification [141]. The conventional collagen extraction method relies on chemical hydrolysis (using acid, alkali, or salt solubilization). Other physical (ultrasound or microwave-based) and/or enzymatic (protease-based) processes could be combined with chemical hydrolysis to enhance the overall extraction [142].

#### 3.2.2. Gelatin

**Figure 8:**
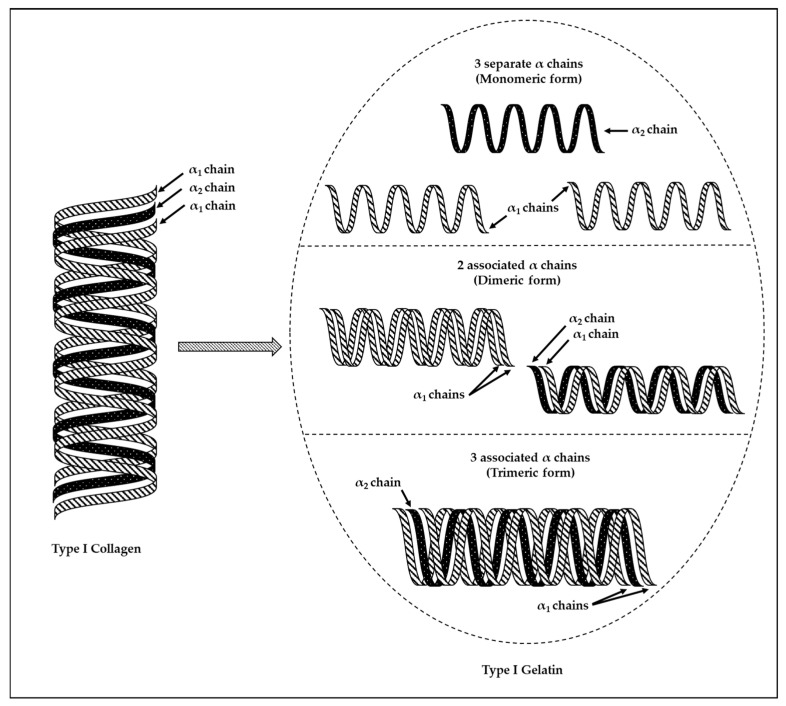
Illustration of the heterogeneous nature of Type I gelatin (figure adapted from Khiari and Gonzalez-Gonzalez [138]).

Gelatin is a natural protein polymer, derived from collagen through hydrolytic degradation involving the breakage of crosslinks between collagen polypeptide chains as well as a partial cleavage of polypeptide bonds [136,143]. Type I gelatin is derived from Type I collagen. The hydrolytic degradation of Type I collagen (which contains two identical α_1_-chains and one α_2_-chain) results in a gelatin mixture comprising either separate (i.e., dissociated) or linked (i.e., associated) α_1_- and α_2_-chains. Therefore, the produced Type I gelatin would be a heterogeneous product with several polypeptide chain combinations (Figure 8). These combinations comprise the monomeric form (three separate chains—two α_1_-chains and one α_2_-chain), the dimeric form (two chains covalently crosslinked or in dimeric form—either two α_1_-chains linked together or one α_1_-chain linked to one α_2_-chain), as well as the trimeric form (three covalently crosslinked chains—two α_1_-chains linked to one α_2_-chain) [138].

In the gelatin extraction process and subsequent to a pretreatment step that removes fat, non-collagenous proteins, and other impurities, the material is treated with either acid or alkaline to partially cleave collagen crosslinks. The choice of acid or alkaline is based on the type and source of the raw material, with alkaline treatment almost exclusively used with bovine raw materials and acid treatment generally used for porcine, poultry, and fish-derived raw materials [138]. Gelatin prepared through the use of acidic treatment is referred to as type A gelatin, while gelatin prepared through the use of alkaline treatment is referred to as type B gelatin [138]. After the pretreatment steps, gelatin is produced through a series of consecutive aqueous thermal extractions carried out at increasing temperatures [144]. The aqueous gelatin solutions are continuously concentrated by evaporation and then filtered, sterilized, and dried. Gelatin produced from animal and fish bones generally follow the same steps as those from skins, except a demineralization operation using a mineral acid is added to remove the minerals. Gelatin derived from bones is referred to as ossein.

## 4. Current Developments and Challenges in the Application of Marine-Derived Biomaterials in Bio-Ink Formulations for 3D Bioprinting

During the past two decades, the development of bio-ink formulations has rapidly increased [145]. A typical bio-ink is made of living cells along with polymers/biomaterials and additives [146]. The majority of bio-inks are hydrogels that support the living cells and possess all the required physicochemical properties for bioprinting (i.e., ideal viscoelastic properties, stability, structural integrity) [147]. The main considerations for the use and development of bio-ink are cost, availability, bioprintability, and biological quality [145].

When it comes to biomaterials used in bio-ink formulations, Pedroza-González et al. [145] recently published the outcomes of a scientometric analysis of 393 original papers published from January 2000 to June 2019 related to the development and use of bio-inks for 3D bioprinting. This analysis indicated that the most popular marine-origin biomaterial (or hydrogel) used in the development of bio-ink formulations is alginate derived from brown seaweed. Gelatin followed by collagen, on the other hand, occupied the second and third places, respectively. Hyaluronic acid also held a place in the top-ten list of the most popular biomaterials in 3D bioprinting [145]. The use of chitosan, carrageenan, chondroitin sulfate, and dermatan sulfate in bio-ink formulations still lingers behind alginate, collagen, and gelatin.

The most important property of alginate is gelation or the ability to form a gel matrix. Alginate hydrogels have the advantages of being natural, non-toxic, biocompatible, and biodegradable [148]. Along with the above-mentioned beneficial characteristics, the success of alginate in bio-ink formulations is mostly attributed to the plasticity of the molecular structure, which is a highly sought-after property in 3D bioprinting [14]. It has been found that 3% alginate-based bio-inks, containing either high molecular weight alginate or a blend with a ratio of 1:2, low molecular weight alginate to high molecular weight alginate, are ideal for the 3D bioprinting of scaffolds with suitable processability and shapes [149]. Alginate (Table 2) has been successfully used as a bio-ink in the 3D bioprinting of vascular tissues [150,151], bone [152,153], skin [154], retina [155], and cartilage [156,157,158].

On the topic of collagen-based bio-inks, Stepanovska et al. [159] recently conducted a systematic search of studies dealing with 3D bioprinting using collagen-based hydrogels, and it was found that Type I collagen-based hydrogels are the most widely utilized. It has also been reported that the printability of collagen bio-ink depends on the kinetics of collagen’s self-organization process into fibrils and the subsequent formation of a hydrogel [160]. However, some studies indicated that the major issue with collagen-based bio-inks is associated with their low mechanical properties [13,161]. A number of approaches have been proposed to enhance collagen’s bioprintability, including the use of supportive hydrogels [162] and strengthening the storage modulus before proceeding with the bioprinting process [163]. It has been recently reported that highly concentrated collagen-based hydrogels (2–3%) could be used as bio-inks without negative effects on cell viability [164]. Collagen (Table 2) has been successfully used as a bio-ink in the 3D bioprinting of bone [165], skin [166,167], and cartilage [168,169].

**Table 2:** Properties and applications of marine-derived biomaterials used in bio-ink formulations for 3D bioprinting.

Biopolymers	Positive Aspects	Negative Aspects	Biomedical Applications
**Alginate**	Natural, non-toxic, biocompatible, and biodegradable	Low mechanical properties	Vascular tissues [123,124], bone [125,126], skin [127], retina [128], and cartilage [129,130,131]
**Gelatin**	Natural, non-toxic, biocompatible, and biodegradable	Low mechanical properties	Bone [143,144], vascularized tissues [145], and cartilage [146,147,148]
**Collagen**	Natural, non-toxic, biocompatible, and biodegradable	Low mechanical properties	Bone [138], skin [139,140], and cartilage [141,142]
**Hyaluronic acid**	Natural, non-toxic, biocompatible, and biodegradable Supports cell growth	Low mechanical properties and slow gelation	Bone [149,150] and cartilage [151]
**Chitosan**	Natural, non-toxic, biocompatible, and biodegradable High mechanical strength	Low thermoplastic characteristics Possible degradation at high temperature	Bone [152,153], skin [154,155], scaffolds suitable for repair of complex structures [156] and cartilage [157]
**Chondroitin Sulfate and Dermatan Sulfate**	Natural, non-toxic, biocompatible, and biodegradable Great stability Low immunogenicity	Possible degradation Low integration with cartilage	Cartilage [158]
**Carrageenan**	Natural, non-toxic, biocompatible, and biodegradable	Brittle and instable	Cartilage tissue engineering [168,169]

Similar to collagen-based hydrogels, gelatin-based hydrogels are non-toxic, biodegradable, and biocompatible, which enable their use as a bio-ink materials in 3D bioprinting. Despite the highly advantageous properties of gelatin-based hydrogels, they still suffer from severe weakness in the mechanical aspects. This challenge is usually overcome in practice through modification with a methacrylate group to create gelatin methacryloyl hydrogels that are currently widely used in bioprinting [170]. It has been suggested that bio-inks based on 5% gelatin solution are suitable for the obtention of 3D constructs [171]. Gelatin (Table 2) has been successfully used as bio-ink in the 3D bioprinting of bone [172,173], vascularized tissues [174], and cartilage [175,176,177].

In comparison with alginate, collagen and gelatin [the most widely used biopolymers/biomaterials in bio-ink formulations], hyaluronic acid has been mostly used in 3D bioprinting as an additive to support cell growth within the hydrogels [14]. This is mainly attributed to the crucial role that hyaluronic acid plays in mediating cellular functions [115]. Some attempts have been made to produce 3D bioprinted bone [178,179] and cartilage [180] using hyaluronic acid-based bio-ink (Table 2). It has been demonstrated that bio-inks based on 5% hyaluronic acid show a desirable shear-thinning property and can successfully be used in 3D bioprinting [179,181].

Chitosan shares similar properties as alginate [natural-origin, non-toxicity, biocompatibility, and biodegradability]. In addition, chitosan has a high mechanical strength and can easily be modified, which could make chitosan-based hydrogels appropriate as bio-ink for 3D bioprinting. However, it has been reported that chitosan is not suitable for thermo-mechanical processing since it does not possess thermoplastic characteristics and can degrade at higher temperature, which could limit its application as a bio-ink [14,182]. According to Zhang et al. [183], the optimum concentrations of chitosan-based bio-inks for bioprinting purposes are in the range of 2.5 to 3%. A lower concentration of chitosan (2%) results in a bio-ink with weaker mechanical properties, while a higher chitosan concentration (>4%) leads to higher cell death [183]. Chitosan (Table 2) has been successfully used as a bio-ink in the 3D bioprinting of bone [184,185], skin [186,187], scaffolds suitable for repair of complex structures [188], and cartilage [189].

Chondroitin and dermatan sulfates have a great practical potential in bio-inks, especially for cartilage regeneration, since they are naturally abundant in cartilage [190]. Both chondroitin and dermatan sulfates share valuable bio-ink relevant properties (Table 2), including greater stability and lower immunogenicity; however, chondroitin and dermatan sulfate-based hydrogels suffer from degradation and do not integrate well with cartilage [191]. Therefore, other compounds are often required in the formulation of chondroitin sulfate and dermatan sulfate-based hydrogels to enhance the bio-ink’s properties as well as its structural stability [190]. For example, both chondroitin sulfate and dermatan sulfate have been combined with alginate-based bio-inks for the 3D bioprinting of cartilage [190].

Carrageenan, similar to alginate, is able to form a gel, and the viscosity of carrageenan gels has been reported to depend on the type, molecular weight, concentration, and temperature [14]. However, it has been found that carrageenan hydrogels are brittle and instable [192]. A number of strategies have been proposed to enhance carrageenan’s bioprintability, including combining carrageenan with other compounds as well as performing various chemical modifications [14]. Marques et al. [193] developed a κ-carrageenan-based bio-ink to bioprint 3D scaffolds and their biocompatibility assays, using L929 mouse fibroblasts, showed high cell viability and attachment. Lim et al. [194] synthesized methacrylated κ-carrageenan, which was subsequently modified through ionic and UV crosslinking to form a hydrogel using NIH-3T3 cells. Bioprinting using this κ-carrageenan-based dual crosslinked bio-ink reduced the shear stress, improved the shape retention capability, and showed good cellular compatibility [194]. Carrageenan (Table 2) has been successfully used to formulate bio-inks for cartilage tissue engineering [195,196].

## 5. Machine Learning in 3D Bioprinting

Designing and characterizing bio-inks and determining their ideal printing conditions are time-consuming and resource-intensive processes, as they are based on empirical experimentations [197,198]. A number of recent studies indicated that machine learning (ML) can assist in formulating the bio-inks and optimizing the bioprinting conditions which, in turn, reduces the number of required experimentations. ML, which is a discipline of artificial intelligence (AI) that focuses on designing systems, has substantially advanced over the past two decades [77,199]. ML is currently a new hot area of research in bioprinting as it offers the possibility to optimize the bioprinting process [200] as well as to predict cell viability [201]. For example, Ruberu et al. [200] explored ML in the quantitative evaluation of the printability and the optimization of 3D extrusion bioprinting using the Bayesian Optimization algorithm. Shi et al. [202] proposed a multi-objective optimization design method for drop-in-demand printing parameters through fully connected neural networks in order to overcome the major issues with this bioprinting process (i.e., satellite generation, very large droplet generation, and very low droplet speed). Shi et al. [203] also investigated a ML technique, named the learning-based cell injection control approach, to improve the efficacy of drop-in-demand printing while automatically eliminating satellite droplets. Reina-Romo et al. [204] evaluated the effect of nozzle shapes and material properties on the shear stress during printing using comprehensive in silico testing. Their results, which were analyzed through a ML approach named Gaussian Process, indicated that shear stress directly affects cell viability [204]. Xu et al. [205] investigated the effect of four stereolithography-based bioprinting parameters (light intensity, exposure time, gelatin methacrylate concentration, and layer thickness) on cell viability. Their results, which were supported by machine learning using a set of algorithms (neural networks, ridge regression, K-nearest neighbors, and random forest), indicated that the developed model was accurate in predicting cell viability [205]. Based on all these studies, ML technology can be considered a powerful tool in advancing further 3D bioprinting. However, the lack and/or the limited availability of bioprinting data remains a major challenge facing the application of ML in 3D bioprinting.

## 6. Conclusion and Future Prospects of Bio-Ink Formulations Using Marine-Derived Biomaterials for 3D Bioprinting

3D bioprinting is a disruptive technology that revolutionized the bio-medical and pharmaceutical fields. However, despite the rapid growth and the remarkable advances, some challenges in using marine-derived bio-ink for 3D bioprinting remain unaddressed or not yet fully solved.

Presently, the inability of bio-inks, formulated using marine-derived biomaterials, to form structurally stable objects should be tackled. In this regard, future research efforts dealing with the development of marine biomaterial-based bio-inks need to focus on strengthening the bio-ink’s mechanical and biological properties (i.e., stabilization of bioprinted structures and the enhancement of cell attachment and proliferation, respectively).

Secondly, limited resolution is still a major challenge in 3D bioprinting. Producing 3D detailed structures using marine biomaterial-based bio-inks is crucial for the widespread application of this technology in the biomedical and pharmaceutical fields, as well as in food industries. Therefore, improving the resolution of the bioprinted constructs need to be considered.

As a concluding remark, 3D bioprinting, using marine biomaterial-based bio-inks, has tremendous opportunities in biomedical, pharmaceutical, and food applications. More specifically, alginate, collagen, and gelatin, which are highly abundant and available in seaweed, fisheries, and aquaculture side-streams, possess a bright future in bio-ink development.

## Figures and Tables

**Table 1:** Properties, advantages, and disadvantages of major bioprinting technologies.

	Bioprinting Technology	Resolution	Cell Viability	Advantages	Disadvantages
**Jetting**	Inkjet	10–100 µm [21,22,23]	70–95% [22,23,24]	Non-contact technique, flexible, low cost, reproducible, and simple [25,26,27]	Thermal and shear damage, non-uniform droplet size, unsuitable for viscous and concentrated bio-inks [25,26,27]
	Laser-Assisted	10–50 µm [28]	80–95% [20,29]	High resolution and cell viability, accurate, suitable for printing of high cell densities and compatible with highly viscous biomaterials [25,26,27]	Often there is difficulty to position the bio-ink to the desired location, low stability, may require further chemical modification [25,26,27]
Extrusion	Pneumatic-, Screw- and/or Piston-Driven	100 µm [30]	40–80% [30,31]	Simple and affordable, good mechanical resistance, possibility to use multiple materials simultaneously [25,26,27,32,33]	Low resolution and cell viability, possible thermal degradation, specific type of material required (thermoplastic) [25,26,27,32,33]
**Vat Polymerization**	Stereolithography (SLA)	20–50 µm [34,35,36,37]	85–95% [38,39]	High resolution, accurate, efficient use of bio-ink, gentler on cells, does not use high temperature and shear stress [40,41,42]	Bioprinted models tend to be fragile, limited availability of bio-ink materials [42]
	Two-Photon Polymerization (TPP)	100 nm to tens of µm [43,44,45]	90–95% [46,47]	Very high resolution and precision, effective in producing 3D micro/nano structures, bioprinting is not limited to the layer-by-layer approach, bioprinting without any geometrical limitations [42,48,49,50,51]	Low throughput, photosensitive materials are very scant, bioprinting process is cumbersome and time-consuming compared to SLA and DLP [42,48,52]
	Digital Light Processing (DLP)	25–50 µm [40,53]	85–95% [54,55]	High resolution, precision, and bioprinting speed, gentler on cells, does not use high temperature and shear stress [40,41,42,54,56,57]	More expensive than SLA, limitation on the size of the finished products, some issues with the photoactive liquid resins used in the printing process (toxicity and odor) [42,56]
